# The Photosystem II Assembly Factor Ycf48 from the Cyanobacterium *Synechocystis* sp. PCC 6803 Is Lipidated Using an Atypical Lipobox Sequence

**DOI:** 10.3390/ijms22073733

**Published:** 2021-04-02

**Authors:** Jana Knoppová, Jianfeng Yu, Jan Janouškovec, Petr Halada, Peter J. Nixon, Julian P. Whitelegge, Josef Komenda

**Affiliations:** 1 Centre Algatech, Laboratory of Photosynthesis, Institute of Microbiology of the Czech Academy of Sciences, Opatovický mlýn, 37981 Třeboň, Czech Republic; knoppova@alga.cz (J.K.); janouskovec@alga.cz (J.J.); 2 Sir Ernst Chain Building-Wolfson Laboratories, Department of Life Sciences, South Kensington Campus, Imperial College London, London SW7 2AZ, UK; j.yu@imperial.ac.uk (J.Y.); p.nixon@imperial.ac.uk (P.J.N.); 3 Laboratory of Molecular Structure Characterization, Institute of Microbiology of the Czech Academy of Sciences, 14220 Praha 4–Krč, Czech Republic; halada@biomed.cas.cz; 4 The Pasarow Mass Spectrometry Laboratory, The Jane and Terry Semel Institute for Neuroscience and Human Behavior, David Geffen School of Medicine, UCLA, Los Angeles, CA 90095, USA; jpw@chem.ucla.edu

**Keywords:** photosystem II, photosynthesis, chlorophyll-binding proteins

## Abstract

Photochemical energy conversion during oxygenic photosynthesis is performed by membrane-embedded chlorophyll-binding protein complexes. The biogenesis and maintenance of these complexes requires auxiliary protein factors that optimize the assembly process and protect nascent complexes from photodamage. In cyanobacteria, several lipoproteins contribute to the biogenesis and function of the photosystem II (PSII) complex. They include CyanoP, CyanoQ, and Psb27, which are all attached to the lumenal side of PSII complexes. Here, we show that the lumenal Ycf48 assembly factor found in the cyanobacterium *Synechocystis* sp. PCC 6803 is also a lipoprotein. Detailed mass spectrometric analysis of the isolated protein supported by site-directed mutagenesis experiments indicates lipidation of the *N*-terminal C29 residue of Ycf48 and removal of three amino acids from the *C*-terminus. The lipobox sequence in Ycf48 contains a cysteine residue at the −3 position compared to Leu/Val/Ile residues found in the canonical lipobox sequence. The atypical Ycf48 lipobox sequence is present in most cyanobacteria but is absent in eukaryotes. A possible role for lipoproteins in the coordinated assembly of cyanobacterial PSII is discussed.

## 1. Introduction

**Figure 1:**
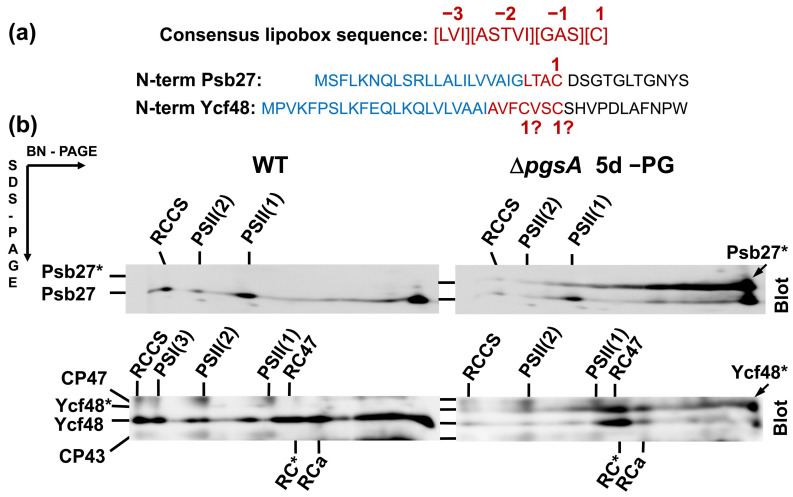
Comparison of the consensus lipobox sequence and *N*-terminal sequences of Psb27 and Ycf48 with designation of signal peptide (blue) and putative lipobox sequences (red) (**a**), and forms of Psb27 and Ycf48 detected in membranes of the *Synechocystis* wild-type (WT) cells and in cells of the Δ*pgsA* mutant after 5 days of phosphatidylglycerol (PG) depletion (**b**). Membrane proteins were separated by 2D BN/SDS-PAGE, the gel was blotted to a polyvinylidene difluoride membrane, and Psb27 and Ycf48 were detected by the specific antibodies. Designation of complexes and proteins: RCCS, supercomplex of PSI and PSII; PSI(3), trimer of photosystem I; PSII(1) and PSII(2), monomeric and dimeric photosystem II; RC47, PSII(1) lacking CP43; Psb27*, and Ycf48*, preprotein versions of both proteins still containing signal peptides for translocation into the lumen. Five micrograms of Chl were loaded for each sample.

During oxygenic photosynthesis, photosystem II (PSII) and photosystem I (PSI) complexes utilize light to extract electrons from water to produce the NADPH and ATP needed for CO_2_ fixation and the synthesis of sugars. The biogenesis of both complexes requires an array of auxiliary protein factors that generally optimize the process, enable the correct binding of cofactors, and protect the assembly intermediates from photodamage [1,2,3,4]. Among them, a group of PSII-specific factors including Psb27 [5], CyanoP [6,7], and CyanoQ [8,9] are typical bacterial lipoproteins (for a review, see [10,11,12]) that bind to the lumenal side of the cyanobacterial thylakoid membrane to stabilize PSII assembly intermediates. Lipoproteins are synthesized as preproteins with a signal peptide targeting them into the lumen. The signal peptide is cleaved within a conserved motif of [LVI]^−3^ [ASTVI]^−2^ [GAS]^−1^ [C]^1^, where [C]^1^ designates the starting cysteine (C) residue of the apoprotein (Figure 1a). This four-residue motif is called the lipobox and is conserved in eubacteria. Prior to cleavage of the signal peptide, the SH group of [C]^1^ is modified by the attachment of a diacylglycerol moiety, which is transferred from phosphatidylglycerol (PG) by preprolipoprotein diacylglyceryl transferase (for a review, see [11]). Finally, the signal peptide is cleaved *N*-terminal of [C]^1^ by signal peptidase II and the released NH_2_ group of [C]^1^ is acylated using lipoprotein *N*-acyltransferase [12].

The Psb27, CyanoP, and CyanoQ lipoproteins are located within the thylakoid lumen, but additional lumenal proteins participate in the biogenesis of both photosystems. One of them is a factor named Ycf48 [13,14], which is required for the efficient insertion of chlorophyll (Chl) into newly synthesized Chl-binding apoproteins of both photosystems [14]. Ycf48 belongs to a family of seven-bladed beta-propeller proteins, which frequently mediate protein–protein interactions [14]. Ycf48 has been detected in cyanobacterial PSII reaction center (RCII) assembly complexes lacking the inner antennae CP43 and CP47 [15] and has been shown to interact with unassembled D1 destined for either de novo assembly of RCII or replacement of damaged D1 during PSII repair [13]. The factor has not been considered a lipoprotein because its signal peptide lacks the canonical lipobox found in most cyanobacteria.

Here, we show by immunoblotting that Ycf48 in a PG-deficient mutant of the cyanobacterium *Synechocystis* sp. PCC 6803 (hereafter *Synechocystis*) is detected as a double band in which the band with lower electrophoretic mobility probably corresponds to the preprotein, suggesting that Ycf48 is also a lipoprotein. This was further supported by a site-directed exchange of the C29 residue for alanine that remarkably weakened the interaction of Ycf48 with the membrane. Finally, the lipidation of Ycf48 was strongly supported by a mass spectrometric analysis of the protein obtained from the isolated RCII complex. Moreover, the protein was found to be shortened by three amino acid residues at the *C*-terminus. The role of lipidation in the function of lumenal PSII assembly factors is discussed.

## 2. Results

### 2.1. Analysis of Ycf48 in PG Deficient Cells

To see the effect of PG limitation on the presence of PSII lipoproteins in *Synechocystis*, cells of a *pgsA* deletion mutant unable to synthesize PG [16] were initially grown in the presence of externally added PG; then, they were washed and transferred into PG-less medium and grown for an additional 5 days. Membranes isolated from these cells were analyzed by two-dimensional blue-native/SDS polyacrylamide gel electrophoresis (2D BN/SDS-PAGE) in combination with Western blotting (Figure 1). In the case of wild-type (WT) cells, the well-characterized PSII lipoprotein Psb27 [5] was detected in monomeric and dimeric PSII complexes and in the unassembled protein fraction, in agreement with [17]. However, in the PG-depleted cells of the PgsA-less mutant, there was an additional larger form of the protein that typically migrated as a large smear across much of the native gel and was not clearly associated with any protein complexes. As the maturation of bacterial prelipoproteins can occur only after the attachment of the diacylglycerol moiety to the C residue of the lipobox, we concluded that the depletion of PG resulted in the accumulation of a fraction of Psb27 as the preprolipoprotein.

When we attempted to detect other lumenal PSII assembly factors on the blot, we found that Ycf48, a well-described PSII assembly factor [13,14], also migrated as two bands. This result raised the possibility that Ycf48 might also be lipidated, even though it lacks the canonical lipobox sequence and had not previously been considered a lipoprotein.

### 2.2. CVSC Motif in the Ycf48 Preprotein May Represent an Alternative Cyanobacterial Lipobox

**Figure 2:**
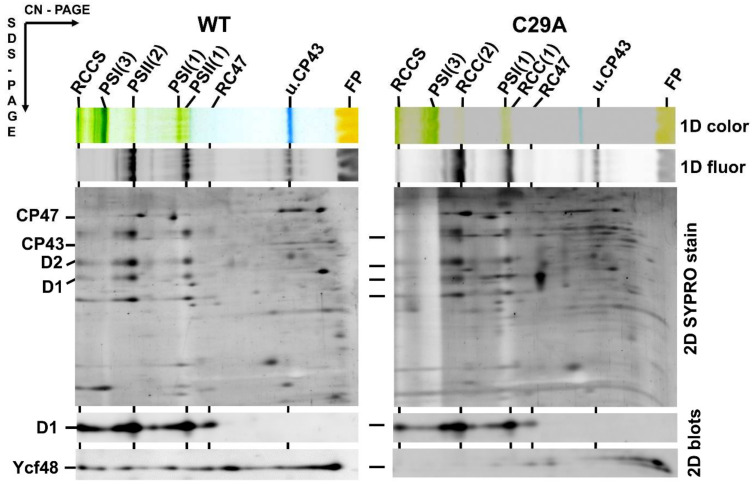
Content of YCF48 in isolated membranes of the *Synechocystis* wild type (WT) and C29A mutant. Membrane proteins were separated by two-dimensional clear-native/SDS polyacrylamide gel electrophoresis (2D CN/SDS-PAGE), and after the first dimension, the gel was photographed (1D color) and scanned for chlorophyll (Chl) fluorescence (1D fluor). After the separation in the second dimension, the gel was stained by Sypro Orange (2D SYPRO stain), blotted to a polyvinylidene difluoride membrane, and immunodecorated using antibodies against D1 and Ycf48 (2D blots). The designation of complexes as in Figure 1, PSI(1) and PSI(3), trimeric and monomeric photosystem I; FP, free pigments. Five micrograms of Chl were loaded for each sample.

**Figure 3:**
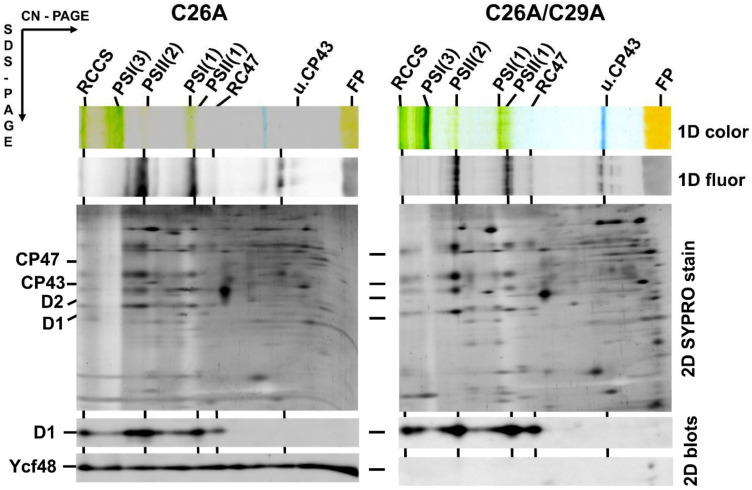
Content of YCF48 in isolated membranes of the *Synechocystis* C26A mutant and C26A/C29A double mutant. Membrane proteins were separated by 2D CN/SDS-PAGE, and after the first dimension, the gel was photographed (1D color) and scanned for Chl fluorescence (1D fluor). After the separation in the second dimension, the gel was stained by Sypro Orange (2D SYPRO stain), blotted to a polyvinylidene difluoride membrane, and immunodecorated using antibodies against D1 and Ycf48 (2D blots). Designation of complexes as in Figure 1 and Figure 2. Five micrograms of Chl were loaded for each sample.

The *N*-terminal amino acid sequence of the Ycf48 preprotein contains two C residues at positions 26 and 29 and thus differs from the sequence of a typical lipobox such as that in Psb27 (Figure 1a). Since it is possible that one of these C residues may nevertheless be lipidated and may represent the initial residue of the mature lipoprotein, we performed site-directed mutagenesis on the *ycf48* gene and replaced either C26 or C29 or both residues with alanine. The membrane and soluble fractions from the obtained mutants, namely C26A, C29A, and C26A/29A, were analyzed by SDS-PAGE. The results showed that in C26A, Ycf48 remained exclusively in the membrane fraction like in WT. In contrast, most of the Ycf48 protein in the C29A and C26A/C29A mutants was in the soluble fraction (Figure S1). We also analyzed the membranes of WT and all mutants using 2D CN/SDS-PAGE (Figure 2 and Figure 3). While Ycf48 of C26A was present in the same complexes as in WT, the residual amounts of Ycf48 found in the membrane fraction of the C29A mutants were mostly located outside of PSII complexes. The presence of the double band of Ycf48 suggests an alternative cleavage site(s) for the protein in the absence of C29. In the C26A/C29A mutant, the small amounts of Ycf48 were again found outside of PSII complexes. In summary, the results were consistent with Ycf48 being a lipoprotein.

**Figure 4:**
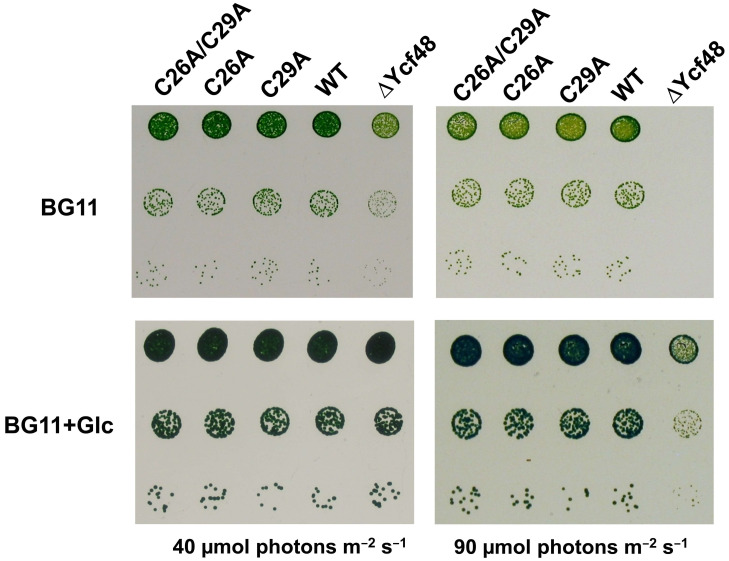
Growth of wild type (WT), C mutants, and Ycf48-less mutant on agar plates under autotrophic (BG11) and photoheterotrophic (BG11+Glc) (40 µmol photons m^−2^ s^−1^) conditions at growth (40 µmol photons m^−2^ s^−1^) and increased (90 µmol photons m^−2^ s^−1^) irradiance. The growth assay was performed as described in [14].

To judge whether the absence of the putative lipid moieties affected the growth of the mutants, we tested their growth rate on agar plates under the normal growth irradiance and at increased irradiance. The results showed that all three site-directed mutants grew similarly to the WT and in this way significantly differed from the Ycf48-less strain and the previously studied mutants affecting the conserved Arg residues important for binding of Ycf48 to Chl-protein complexes (Figure 4; [14]). Thus, lipidation, which is predicted to occur in only certain cyanobacterial species (see below), is not critical for the function of the protein under the growth conditions tested.

### 2.3. Mass Spectrometric Analysis Confirmed the Presence of a Lipidic Moiety at the N-Terminus of the Ycf48 Protein and Shortening of the C-Terminus

**Figure 5:**
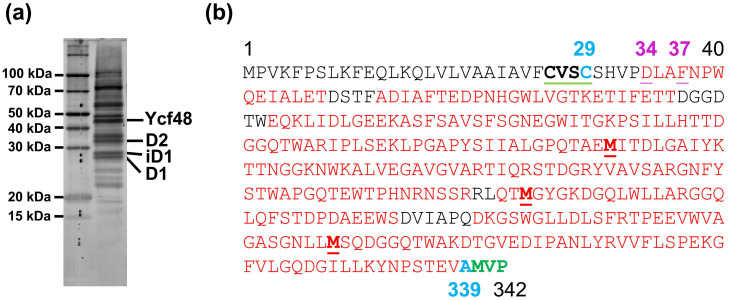
Electrophoretic analysis of the preparation isolated from the strain expressing His-D2 and lacking CP47 (**a**) and sequence of the Ycf48 with designated crucial residues and coverage obtained by its MS bottom–up analysis (**b**). (**a**): The preparation was analyzed by SDS-PAGE, and after staining with Coomassie, the bands of Ycf48, D2, and both forms of the D1 protein were identified using MS. (**b**): The part of Ycf48 sequence identified after in-gel digestion using chymotrypsin and/or Asp-N protease is depicted in red letters. The designated amino acid residues are: (i) C29 residue (in blue) assumed to be the initial *C* residue of the putative lipoprotein; (ii) D34 residue (numbered and underlined in violet) identified as the most *N*-terminal residue in the Asp-N fragments; (iii) F37 residue (numbered and underlined in violet) annotated as the initial protein residue in SwissProt (P73069); M151, M288, and M224, cleavage sites by cyanogen bromide (in bold and underlined), and (iv) A339 residue (in blue) as the last residue found in the both chymotrypsin and Asp-N fragments. The non-canonical lipobox CVSC is designated with bold letter and green underline, putative cleaved MVP sequence is in green.

The characterization of the C29 mutants strongly supported the existence of lipidation at the *N*-terminus of the mature Ycf48 protein. In order to obtain conclusive evidence, we extensively characterized the protein using MS techniques. Since the protein is present in WT membranes at low levels, we took advantage of an Ycf48-enriched preparation obtained from a mutant expressing His-tagged D2 protein and lacking CP47. This strain accumulates an increased amount of PSII assembly intermediates consisting of D1, D2, and Ycf48 as the main protein components (Figure 5a; see also [18]). This preparation was analyzed by SDS-PAGE, and the stained band of Ycf48 was subjected to digestion with chymotrypsin and Asp-N protease followed by MALDI MS analysis (Figure 5b). Using this approach, we were able to cover most of the protein sequences starting from the D34 residue at the *N*-terminus. However, we could not identify any *N*-terminal peptide fragment preceding D34. Nevertheless, this experiment confirmed that a cleavage site at F37 as predicted in the Swiss-Prot database is incorrect. However, there was indication for another post-translational modification. As the last residue identified in both chymotrypsin and Asp-*N* fragments was A339, we conclude that the last three amino acid residues MVP are most probably lacking.

**Figure 6:**
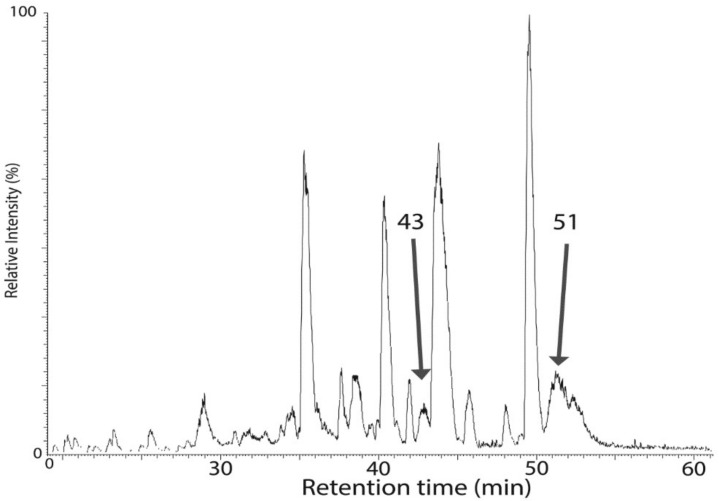
Analysis of the His-D2/ΔCP47 preparation using a reverse-phased HPLC column PLRP/S in combination with high resolution (HR) mass spectrometry. The separation was performed at 40 °C, and elution was performed using a gradient from 0.1% trifluoroacetic acid (TFA) in water to a mixture (50/50) of acetonitrile containing 0.1% TFA and isopropanol. The eluted protein fractions were analyzed by offline nanospray HR MS, and species eluted at 43 and 51 min, which were found to contain Ycf48 by bottom–up MS, were selected for further analysis.

To obtain more detailed information about the nature of Ycf48 post-translational modification (PTM), we used a top–down approach in combination with high-resolution MS analysis. For this purpose, the whole His-D2 preparation was analyzed using reversed-phase chromatography and the eluted proteins were subjected to high-resolution MS identification. The chromatogram (Figure 6) consisted of a large number of peaks, with the peak eluting at 51 min and showing an average mass of 34,689.12 Da ascribed to Ycf48 after peak collection and bottom–up analysis of its components. However, the determined mass did not agree with the predicted mass based on a single cleavage of the signal peptide at F36. Instead, the difference of 815 Da between the predicted and measured masses of Ycf48 (Table 1) is consistent with cleavage of Ycf48 after the A339 residue (to remove the last three residues) and before the C29 residue, and subsequent modification of the SH group of C29 with diacylglycerol and acylation of the NH_2_-group.

**Figure 7:**
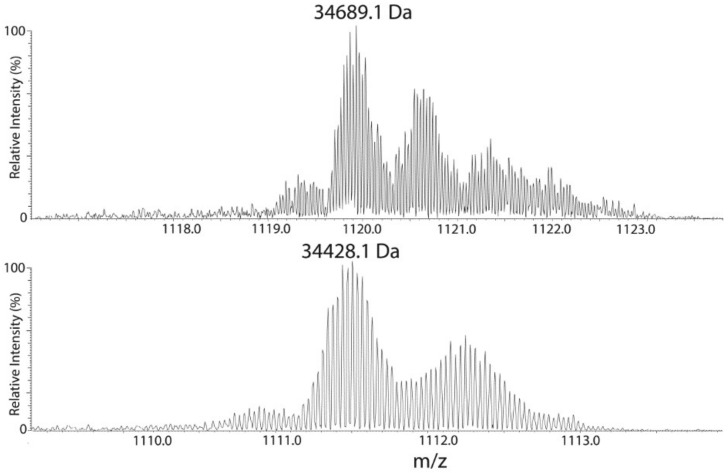
Heterogeneity of His-D2/Δ CP47 protein species eluted from a RP HPLC PLRP/S column at 43 and 51 min. The 51 min fraction contains a modestly heterogeneous protein of average mass 34,689 Da, and other species differing in fatty acid chain lengths. A less hydrophobic protein of 34,428 Da eluted at 43 min protein is less heterogeneous. The difference of 261 Da between both proteins corresponds to the loss of an 18:2 or 18:3 fatty acid from the 34,689 Da species.

Interestingly, a second peak eluting at about 43 min also contained Ycf48 as judged from the bottom–up analysis, and the mass of its major species differed from the 51 min species by a mass of 261 Da (Table 1), which corresponds to a loss of an 18-carbon fatty acid chain carrying one or two double bonds. Whether this fatty acid was lost from the NH_2^−^_ or the SH-group of C29 is unclear, but it reduced the retention time in HPLC by some 8 min, and on closer inspection, the peak at 43 min revealed a lower heterogeneity compared with three fatty acid-containing native 51 min species (Figure 7).

Heterogeneity is typical of lipidated proteins, with the predominant species dependent upon growth conditions. In the 51 min species, we saw a small population that appeared to have a fatty acid with two fewer carbons (−28 Da; C_2_H_4_) and another population that is similarly bigger (+28 Da; C_2_H_4_), and so on. There is probably also some double bond heterogeneity, but this is hard to resolve, because it splits the isotope envelope by just 2 Da for each bond (2 Da; 2H). In the experiments performed, the mass spectrometer did not distinguish positional isomers. The net result is that the exact hydrophobicity was more variable than single protein species, broadening the chromatographic peak, although no differences were noted across the peak. The very high retention time of this protein at 51 min was consistent with the hydrophobicity resulting from lipidation.

Further confirmation of Ycf48 PTM was obtained using cyanogen bromide treatment of the 51 min HPLC fraction and a top–down MS analysis of the obtained fragments. Since the Ycf48 amino acid sequence contains just three methionines (Figure 5b), only a limited number of peptides could be theoretically obtained. We detected two peptides with masses 14,117 and 22,188, corresponding to the putative *N*-terminal fragments C29-M151 and C29-M224, respectively, but the difference from the theoretical masses (taking into account a possible oxidation of 151 in the latter fragment) was in the range of 813-816, which again agrees with addition of *S*-diacylglycerol plus an NH_2_-bound fatty acid (Table 2). The *C*-terminal peptide 289–339 has a mass of 5525.3 Da consistent with cleavage after M288 and a *C*-terminus lacking the last three amino acids MVP. The lack of residual homoserine (or its lactone) on the *C*-terminal CNBr fragment proves the absence of MVP in the mature protein. The inhibited cleavage at M288 due to its oxidation to the sulfoxide and cleavage at M224 gave rise to an observed fragment 225–339 with mass of 12,505.0 Da.

Overall, our analyses proved the modification of the *N*-terminus of Ycf48 and absence of the tripeptide MVP at the *C*-terminus. Based on the mass difference of 813, we propose that Ycf48 is lipidated at the *N*-terminal C29.

### 2.4. Comparing Lipoboxes and N- and C-Termini of Ycf48 among Cyanobacteria and Chloroplasts

**Figure 8:**
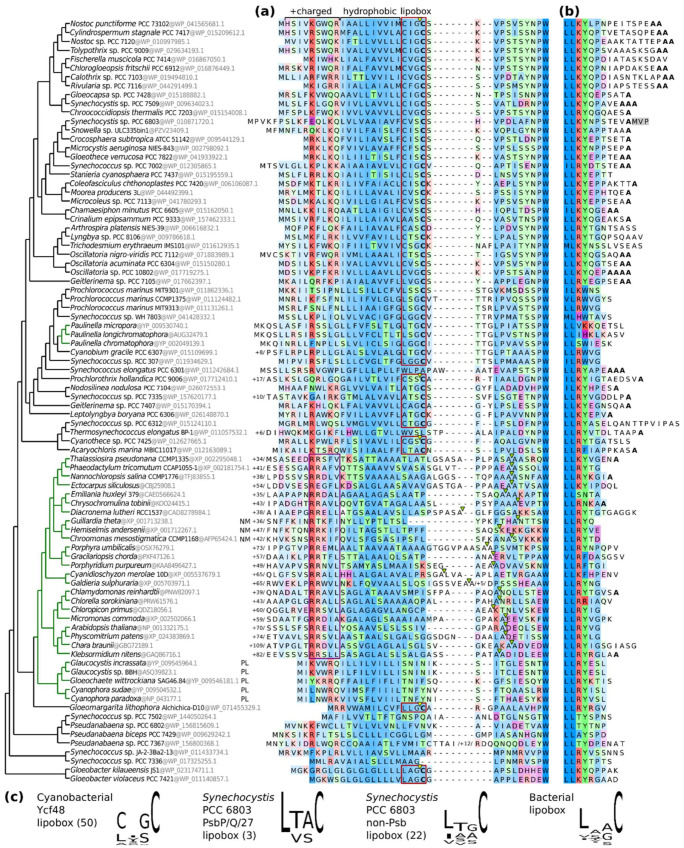
The structure of Ycf48 termini. Ycf48 sequences from 57 cyanobacteria and 31 plastids (green tree branches) are aligned and ordered by their relationship (left). Sequence residues are colored according to the amino acid type. Alignment sites are shaded by their conservation. Plastidial proteins are encoded in the nuclear genome except for the glaucophytes (plastid genome = PL) and cryptomonads (nucleomorph of the endosymbiont = NM). (**a**) The *N*-terminus of Ycf48. Cyanobacterial signal sequences contain positively charged and hydrophobic regions (top) and predicted lipoboxes (red boxes) with signal cleavage sites (yellow triangles). Plastid signal sequences include the thylakoid lumenal transfer peptide with a twin-arginine targeting (TAT) motif (purple box) and predicted cleavage sites (light green tringles). The number of residues that have been trimmed to the left of sequences or within them is indicated. (**b**) The *C*-terminus of Ycf48. Terminal alanine residues highlighted in bold. Residues that are cleaved off in Ycf48 of *Synechocystis* sp. PCC 6803 are shown on the gray background. (**c**) Sequence logos of lipobox motifs. The number of sequences used for making the logos is in round brackets. Ycf48 lipoboxes correspond to red boxes in (**a**). *Synechocystis* and bacterial lipoprotein set were compiled from the DOLOP database and [20].

The lipidation of Ycf48 in *Synechocystis* in the absence of a canonical lipobox prompted us to check for the presence of Ycf48 lipoboxes in other cyanobacteria, algae, and plants (Figure 8). The analysis revealed that Ycf48 in most cyanobacteria contains a special type of lipobox that predominantly starts with C. This is especially apparent in the evolutionarily younger group of core cyanobacteria (top of Figure 8). However, some cyanobacteria including the evolutionary oldest *Gloeobacter* lineage contain the standard lipobox, and others lack C at their *N*-termini altogether and therefore are most probably not lipidated (for instance, *Thermosynechococcus elongatus*). The absence of lipoboxes also applies to Ycf48 homologues in photosynthetic eukaryotes, which are mostly encoded in the nucleus (a notable exception to this are the glaucophytes and cryptomonads where Ycf48 remains encoded in the chloroplast genome and in the nucleomorph of an endosymbiont, respectively; see Figure 8). A comparison of the complete *N*-termini also shows that cyanobacterial Ycf48 is translocated to the thylakoids via a SecA-mediated pathway whereas chloroplastic Ycf48 is translocated via a TAT-mediated mechanism requiring a long hydrophobic peptide and double Arg motif, see [19]. The *C*-terminal Ycf48 residue in the core cyanobacteria is predominantly alanine, such as in *Synechocystis*, but the residue varies in earlier-branching taxa. It cannot be excluded that some of these *C*-termini might be processed as identified here in *Synechocystis*. There are several proteases in the lumen of cyanobacteria that might catalyze such a cleavage.

## 3. Discussion

Three lumenal accessory factors involved in the assembly and maintenance of cyanobacterial PSII have been identified as lipoproteins [10]. They include Psb27, CyanoP, and CyanoQ, which is found in oxygen-evolving PSII complexes but could also act as an assembly factor during the later stages of PSII biogenesis [21]. Our MS analysis showed that Ycf48 represents another member of this cyanobacterial lipoprotein family. Although these lipoproteins seem to be conserved in cyanobacteria, the corresponding homologues in higher plants most probably do not contain lipidic groups. Previous biochemical data support the idea that Ycf48 acts early in PSII assembly and binds to unassembled precursor D1, which then associates with the PsbI subunit and Ycf39 factor, forming the so-called D1 assembly module [2,18]. In plants, the newly synthesized D1 protein was proposed to be palmitoylated [22], and perhaps this PTM could have a similar function to the lipidation of Ycf48. During PSII assembly in *Synechocystis*, Ycf48 attached to the D1 assembly module is thought to interact with CyanoP bound to the D2 module to help form the RCII complex [7]. The subsequent formation of larger PSII complexes is dependent on the synthesis of CP47, which is thought to be promoted by PAM68, which is a membrane protein that interacts with Ycf48 [23,24]. The seven-bladed protein structure of Ycf48 [14] is well suited for mediating the multiple interactions needed to promote efficient assembly. The detection of Ycf48 in larger PSII core complexes might reflect retention of Ycf48 on the lumenal surface of PSII prior to light-driven assembly of the oxygen-evolving complex [14].

In *E. coli*, transfer of the diacylglycerol moiety from a phospholipid to the *N*-terminal C residue of the preprolipoprotein is catalyzed by Lgt (preprolipoprotein diacylglycerol transferase, P72846) and the genome of *Synechocystis* indeed encodes one similar protein (48% identity, gene *sll1187*). This enzyme should be responsible for recognition of the lipidation site, which is dependent on the presence of a lipobox.

However, the lipidated C29 residue in *Synechocystis* Ycf48 is preceded by a CVS triad, of which C26 at the -3 position does not meet the requirements for a canonical lipobox. A cyanobacteria-wide comparison of Ycf48 sequences showed that most species contain a second *C* residue in the -3 position rather than L/V/I found in typical lipoboxes. Nevertheless, the replacement of C26 by alanine did not result in any detectable change in Ycf48 abundance and behavior, suggesting that normal lipidation of Ycf48 occurred in this strain and that the specificity of the diacylglycerol transferase is rather low. To our knowledge, this is the first identification of an *N*-terminal *C*-lipidation in a bacterial protein that does not require the strict presence of a canonical lipobox. Moreover, our bioinformatics analysis demonstrates that *C* at the −3 position is present in the Ycf48 proteins of most core cyanobacterial species. In contrast, the remaining PSII-related lipoproteins as well as the other cyanobacterial lipoproteins (Figure 8c) contain the canonical lipobox, and therefore, it is not clear why Ycf48 is so exceptional in this respect.

As is evident from Figure 1b, the Psb27 preprotein does not bind to PSII, indicating that lipidation occurs extremely quickly after translocation of the protein, before Psb27 can bind to CP43 or PSII. On the other hand, a substantial fraction of the Ycf48 preprotein is bound to RCII complexes, suggesting that the lipidation of Ycf48 is slower and might occur when the protein is already bound to the D1 protein. We speculate that the signal peptide of the preprotein may help target Ycf48 into a membrane region in which the D1 protein is specifically synthesized (for instance, the PratA-derived membranes [3]), and therefore, it is advantageous to slow down its cleavage via slowing down the lipidation events. The lack of binding of Ycf48 to PSI trimers (Figure 1b, for interaction of Ycf48 with PSI see [14]) is in agreement with this hypothesis.

The role of the lipid moiety in the function of Psb27, CyanoP, CyanoQ, and Ycf48 is not clear, although Juneau et al. have concluded that lipidation may stabilize the CyanoQ protein [25]. Since PSII biogenesis seems to occur in specific regions of the thylakoid membranes, lipids may help maintain the location of assembly factors within these regions. The existing data suggest that each of the lipoproteins preferentially interacts with one of the large Chl-binding proteins of PSII: Ycf48 with D1 [13], CyanoP with D2 [7], Psb27 with CP43 [17], and perhaps CyanoQ with CP47 [26]. Lipidation allows tethering of the protein to the membrane whilst permitting a protein to rotate, which might be important for a more efficient access to interacting proteins close to the membrane surface. We hypothesize that the PSII lipoproteins could also form a 3D network at the lumenal surface of the biogenesis regions that directs newly synthesized Chl-binding proteins to the appropriate location and thereby optimizes assembly. In addition, these lipoproteins might keep the nascent, not fully functional PSII, within the biogenesis region until oxygen evolution is activated and the assembly factors are replaced by the lumenal PSII subunits PsbO, PsbV, and PsbU.

## 4. Materials and Methods

### 4.1. Synechocystis Strains, Their Construction and Growth Conditions

The ΔPgsA strain characterized in [16] was kindly provided by Z. Gombos, BRC HAS, Szeged, Hungary. The ΔYcf48 mutant was prepared by replacing the *ycf48* gene with a kanamycin-resistance cassette as previously described in [14]. The Ycf48 C26/29A single and double mutations were introduced using transformation vectors based on the pYcf48WTgent plasmid [14] as a template. Overlap extension PCR reactions were carried out using the primer set Ycf48-KasI-F and Ycf48C26A-2R or Ycf48C29A-2R or Ycf48C26/29A-2R and primer set Ycf48C26A-3F or Ycf48C29A-3F or Ycf48C26/29A-3F and Ycf48-HindIII-R (Table 2). Then, the resulting two fragments for each construct were PCR fused with the primer set Ycf48-KasI-F and Ycf48-HindIII-R and integrated into pYcf48WTgent plasmid via KasI and HindIII sites through In-Fusion cloning (Takara Bio, Mountain View, CA, USA), replacing the wild-type sequence from the vector. Then, the resulting plasmids pYcf48C26Agent, pYcf48C29Agent, and pYcf48C26/29Agent were used to transform the ∆Ycf48 strain to yield the C26A, C29A, and C26/29A mutants.

The strain expressing a His-tagged version of D2 and lacking CP47 (His-D2/∆CP47) was derived from the Tol145 mutant [27] lacking both copies of D2. Plasmid pDC074 carrying a kanamycin-resistance cassette [28] was used as parental vector for D2 mutagenesis. The coding sequence of the 6xHis tag was introduced after the START codon of *psbDI* gene by overlap extension PCR using the primer sets psbDC1F/psbD-His-2R and psbD-His-3F/psbDC4R (Table 2). Then, the plasmid was used to transform the Tol145 strain, yielding the His-D2 mutant. Then, the *psbB* gene was disrupted using the pPsbB-gent vector where *psbB (slr0906)* was replaced with a gentamycin-resistance cassette.

The strains were grown photoautotrophically in liquid BG-11 medium on a rotary shaker at moderate (normal) light intensities (40 µmol photons m^−2^ s^−1^) at 28 °C. For the preparation of the Ycf48-enriched PSII assembly intermediate from the His-D2/∆CP47 strain, four liters of the liquid cell culture were grown in a 10-litre round-bottomed flask in BG11 medium supplemented with 5 mM glucose at 28 °C at a surface irradiance of about 100 µmol photons m^−2^ s^−1^. The culture was agitated using a magnetic stirrer and bubbled with air. For the growth assay, cell cultures were spotted on BG-11 plates and grown either autotrophically or mixotrophically at normal (40 µmol photons m^−2^ s^−1^) or increased (70–90 µmol photons m^−2^ s^−1^) irradiance.

### 4.2. Determination of Chlorophyll Content

To determine chlorophyll content, pigments were extracted from cell pellets with 100% methanol, and the chlorophyll concentration was determined spectroscopically [29].

### 4.3. Preparation of Cellular Membranes and His-tag Purification

Cell cultures were harvested at OD_750nm_ ≈ 0.5–0.7. Cells were pelleted, washed, and resuspended in buffer B (25 mM MES/NaOH, pH 6.5, 10 mM CaCl_2_, 10 mM MgCl_2_, 25% glycerol). Cells were broken using zirconia–silica beads in a mini bead-beater, and the membrane and soluble fractions were separated by centrifugation at 36,000× *g* for 20 min. Then, the membranes were resuspended in buffer B.

To obtain the Ycf48-enriched preparation, thylakoid membranes were isolated in buffer B containing EDTA-free protease inhibitor cocktail (Sigma-Aldrich, St. Louis, MI, USA) and then solubilized with 1.5% (*w*/*v*) n-dodecyl-β-d-maltoside (β-DDM) under slow rotation (60 min, 10 °C), and the insoluble fraction was separated by centrifugation (43,000× *g*/25 min, 4 °C). Approximately 7 mL of solubilized material containing 7 mg Chl was loaded onto a column containing 1 mL of Ni-NTA agarose resin (MACHEREY-NAGEL, Dueren, Germany) pre-equilibrated with buffer B containing 0.04% DM (wash buffer, WB). The flow-through was collected and loaded repeatedly 5 times. The resin was first washed 3 times with two volumes (V) of WB (3 × 2 V), then successively with WB containing increasing concentrations of imidazole (IM) according to the following scheme: 3 × 2 V (10 mM IM), 2 × 2 V + 1 × 1 V (20 mM IM), 1 × 1 V + 1 × 0.5 V (30 mM IM), and then again with 1 × 3 V of WB. After the washing steps, the resin was incubated for 5 min with 0.5 V of elution buffer (WB plus 200 mM IM, pH 8), transferred into centrifuge filter tube, and spun down (400× *g*/2 min, 4 °C) to get the eluate.

### 4.4. Protein Electrophoresis and Immunoblotting

For native electrophoresis in the first dimension, isolated cellular membranes were solubilized with β-DDM (ratio to Chl = 60 (*w*/*w*)) and then separated using blue native [30] or clear native [31] polyacrylamide gel electrophoresis (BN/CN-PAGE) on a 4–12% (*w*/*v*) gradient gel. Individual components of the protein complexes were resolved by incubating the native gel strip in a Tris-HCl buffer (pH 7) containing 1% (*w*/*v*) sodium dodecyl sulfate (SDS) and 1% (*w*/*v*) dithiothreitol (DTT) for 30 min at room temperature, and proteins were separated in the second dimension by SDS-PAGE in a denaturing 12–20% polyacrylamide gel containing 7 M urea [32]. For standard single dimension SDS-PAGE, membrane proteins were solubilized with 1% (*w*/*v*) SDS and 2% (*w*/*v*) DTT for 30 min at room temperature before loading onto the gel. Proteins were either stained with Coomassie blue, or by Sypro Orange. In the latter case, they were subsequently electroblotted onto a polyvinylidene difluoride membrane. Membranes were incubated with specific primary antibodies and then with a secondary antibody conjugated with horseradish peroxidase (Sigma-Aldrich, St. Louis, MI, USA). The following primary antibodies were used in the study: anti-Ycf48 raised in rabbit against recombinant *Synechocystis* Ycf48 [14]; anti-Psb27, anti-CP47, anti-CP43, and anti-D1 [17].

### 4.5. Mass Spectrometry Analyses

Enzymatic digestion of the Ycf48 gel band was performed by chymotrypsin and Asp-N protease as described in [33]. MALDI spectra were measured using SolariX XR™ FT-ICR mass spectrometer (Bruker Daltonics, Billerica, MA, USA) operating with mass accuracy better than 3 ppm. The identity of *N*-, *C*-terminal, and protease-unspecific fragments was verified using MS/MS spectra obtained on an Ultraflex III MALDI-TOF instrument (Bruker Daltonics, Bremen, Germany). The mass spectra were searched against SwissProt database subset of *Synechocystis* proteins using an in-house MASCOT 2.3 search engine.

For intact protein analysis, the isolated His-D2 preparation was precipitated at −20 °C with 80% acetone as described in [34] and dissolved in 70% aqueous formic acid prior to analysis by HPLC within 2 min. A column (PLRP/S; 2 × 100 mm; 5 µm bead, 300 Å pore size; Agilent, Santa Clara, CA, USA) at 40 °C was equilibrated at 120 µL/min in 95% buffer A and 5% buffer B. Buffer A was 0.1% trifluoroacetic acid (TFA) in water, and buffer B was a 50/50 mixture of acetonitrile/isopropanol containing 0.05% TFA. A gradient of increasing B was applied (5 to 95% B over 70 min). The column eluate was split, and half was directed to an ion-trap mass spectrometer (LTQ, Thermo Fisher, Waltham, MA, USA) operated using a standard electrospray source in positive ion mode (instrument parameters were as used for instrument calibration). The remaining 50% was directed to a fraction collector (1 min fractions). Data were analyzed with the assistance of deconvolution MagTran software [35].

The identification of Ycf48 in the fractions was performed using static nanospray infusion on a hybrid ion trap 7 Tesla Fourier-transform ion cyclotron resonance mass spectrometer (LTQ FT Ultra; Thermo Fisher). A small volume (5–10 µL) of sample previously purified by HPLC (0.1% trifluoroacetic acid—TFA, 75% acetonitrile/isopropanol) was loaded into a spray needle using a 10 µL syringe. Immediately prior to MS, the tip was gently broken, removing the finest part of the capillary. For positive ion analysis, as used in this study, a voltage between 1300 and 1500 V was used for nano-electrospray ionization, giving a final spray flow of 50 nL/minute. Data were analyzed with the assistance of deconvolution MagTran software [35].

For cyanogen bromide mapping of Ycf48, 2 µL of a 1 g/mL solution of CNBr (99.995% purity; Sigma-Aldrich, St. Louis, MI, USA) in acetonitrile was added to a similar HPLC fraction as above (20 µL). After incubation at 24 °C in the dark for 5 h, the sample was analyzed by HPLC as described above. The eluate line was directed fully for MS. Data were analyzed with the assistance of deconvolution MagTran software [35].

### 4.6. In Silico Analysis of Ycf48 Sequences

The *Synechocystis* Ycf48 sequence was used as a query to mine homologues in representative cyanobacterial and eukaryotic genomes by BLASTP homology searches. Orthologous sequences (a single variant per genome was identified) were aligned by using the localpair algorithm in MAFFT v. 7.471 [36] and gaps in the flanking regions were compressed manually for visual clarity. Lipoprotein signal and thylakoid transfer peptides with their most probable cleavage sites were predicted in LipoP v. 1.0 and TargetP v. 2.0, respectively. Sequence logos were created in Jalview v. 2.11.1.3. The *Synechocystis* and bacterial lipoboxes used to make the logos were compiled from the DOLOP database and published residue distribution [20], respectively. Species relationships in Figure 8 are derived from published phylogenomic studies (primarily [37]).

## Figures and Tables

**Table 1:** MS analysis of the intact Ycf48 and its fragments after cyanogen bromide cleavage. Cleavage of the protein after methionine residues resulted in the generation of fragments that were detected by MS. The results confirmed the putative *N*-terminal modification with a PTM involving the addition of 813–816 (or 553) and cleavage of the last three amino acid residues. *N*-terminal fragments were identified as homoserine lactones and +16.0 stands for methionine oxidation.

Sample	Retention Time (min)	Measured Mass (Average, Da)	Calculated Mass (Average, Da)	Assignment (From Init Met)	Delta (Da)
**Intact Protein**					
Intact protein	51	34,689.1	33,874.6	29–339	814.5
Intact protein	43	34,428.1	33,874.6	29–339	553.5
**CNBr Fragments**					
*N*-terminal fr	59.9–60.2	14,117.0	13,303.6	29–151	813.3
	58.4–58.6	22,188.5	21,356.6 + 16.0	29–224	815.8
*C*-terminal fr	28.3	5525.3	5525.1	289–339	0.1
	32.7	12,505.0	12,487.8 +16.0	225–339	1.1

**Table 2:** Primers used for Ycf48C26/29A and His-D2 mutations. Modified codons and *His*-tag are underlined, unchanged C codons in Ycf48 positions 26 or 29 are in bold.

Primers	Sequences (5′–3´)
Ycf48-KasI-F	ATGTCCGGTGTGTGGCGCC
Ycf48-HindIII-R	TACGGCCCCCTCCACCAAAGCTT
Ycf48C26A-3F	GCGGCGATCGCCGTTTTCGCGGTGAGCTGCAGCCATGTGCCGGA
Ycf48C26A-2R	TCCGGCACATGGCTGCAGCTCACCGCGAAAACGGCGATCGCCGC
Ycf48C29A-3F	TCGCCGTTTTCTGTGTGAGCGCGAGCCATGTGCCGGATTTGGCCT
Ycf48C29A-2R	AGGCCAAATCCGGCACATGGCTCGCGCTCACACAGAAAACGGCGA
Ycf48C26/29A-3F	GCGGCGATCGCCGTTTTCGCGGTGAGCGCGAGCCATGTGCCGGATTTGGCCT
Ycf48C26/29A-2R	AGGCCAAATCCGGCACATGGCTCGCGCTCACCGCGAAAACGGCGATCGCCGC
psbDC1F	AGTTGCGACAAAATAACCCAGCTCCAGCAA
psbD-His-2R	GCGCGTCCGACTGCAATAGTATGATGATGATGATGATGCATAAATGCAAATCCTCTTGCGTAGCT
psbD-His-3F	AGCTACGCAAGAGGATTTGCATTTATGCATCATCATCATCATCATACTATTGCAGTCGGACGCGC
psbDC4R	TTGCCAAAGTATTCTCCTGATTTAAATGATATTGAGCA

## Data Availability

Not applicable.

## Supplementary Materials

The following are available online at https://www.mdpi.com/article/10.3390/ijms22073733/s1, Figure S1: Analysis of membrane and soluble fractions isolated from cells of wild type (WT) and C26A, C29A and C26A/C29A mutants.
