# Supplementary material for: The Photosystem II Assembly Factor Ycf48 from the Cyanobacterium Synechocystis sp. PCC 6803 Is Lipidated Using an Atypical Lipobox Sequence
Source: Int J Mol Sci. 2021 Apr 2;22(7):3733. doi: 10.3390/ijms22073733 (PMC8038367; doi:10.3390/ijms22073733)
Supplement: Supplementary file 1 [file ijms-22-03733-s001.pdf]

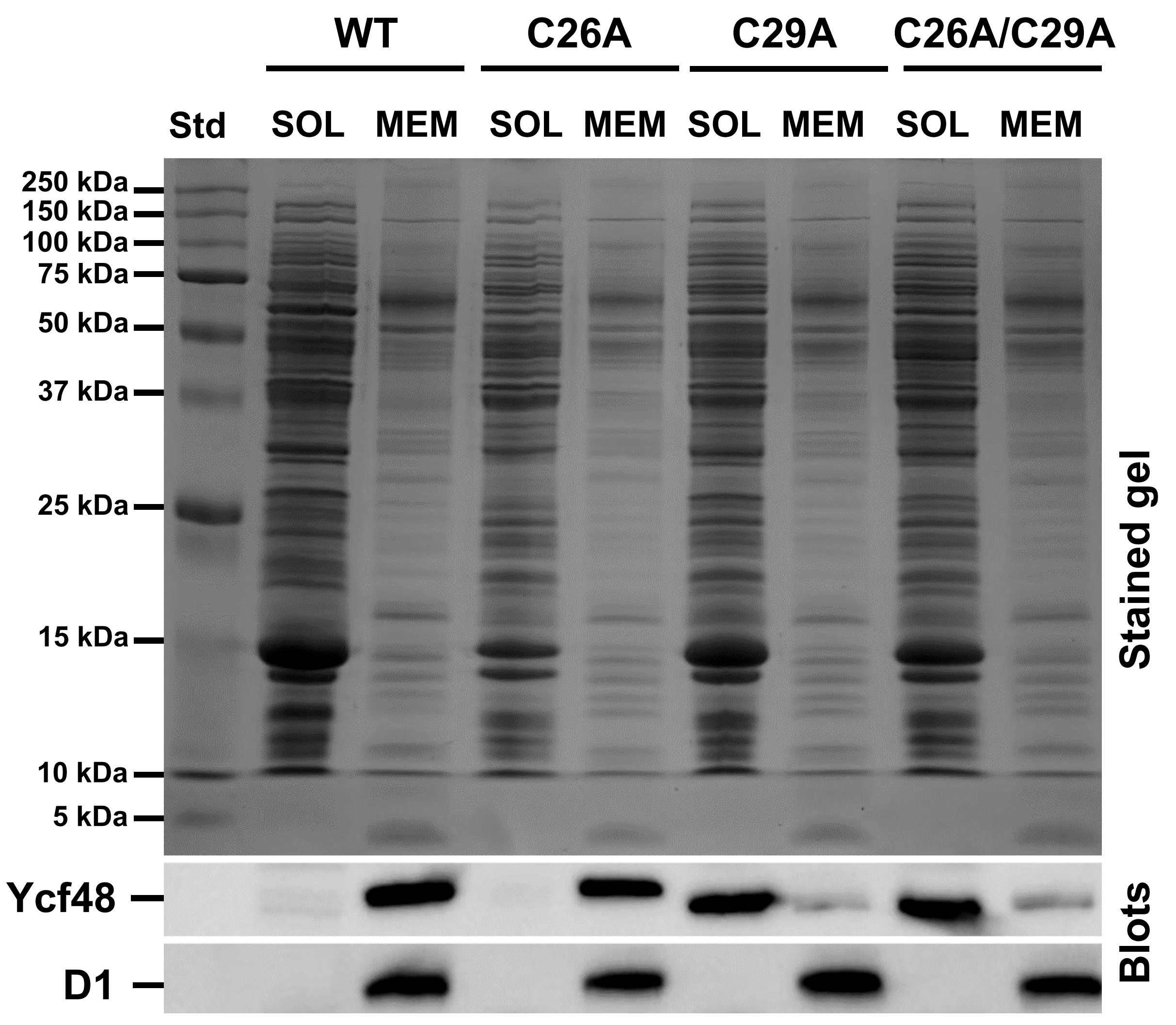

Fig. S1. Analysis of membrane and soluble fractions isolated from cells of WT and Cys26A, Cys29A and Cys26A/Cys29A mutants. The membrane (MEM) and soluble (SOL) fractions were analysed using SDS PAGE, proteins electroblotted and Ycf48 and D1 were detected using specific antibodies. 1 μg of Chl per membranes and corresponding amount of the soluble fraction were loaded for each sample.
